# Pathology of porcine peripheral white blood cells during infection with African swine fever virus

**DOI:** 10.1186/1746-6148-8-18

**Published:** 2012-02-28

**Authors:** Zaven Karalyan, Hovakim Zakaryan, Hranush Arzumanyan, Khachik Sargsyan, Henrik Voskanyan, Lina Hakobyan, Liana Abroyan, Aida Avetisyan, Elena Karalova

**Affiliations:** 1 Laboratory of Cell Biology, Institute of Molecular Biology of NAS, 7 Hasratyan St, 0014 Yerevan, Armenia; 2 Scientific Center of Stock Breading and Veterinary RA, Nubarashen Sarahart, Yerevan 0071, Armenia

## Abstract

**Background:**

African swine fever virus (ASFV) is the causative agent of African swine fever (ASF) that is the significant disease of domestic pigs. Several studies showed that ASFV can influence on porcine blood cells in vitro. Thus, we asked ourselves whether ASFV infection results in changes in porcine blood cells in vivo. A series of experiments were performed in order to investigate the effects of ASFV infection on porcine peripheral white blood cells. Nine pigs were inoculated by intramuscular injection with 10^4 ^50% hemadsorbing doses of virus (genotype II) distributed in Armenia and Georgia. The total number of fifteen cell types was calculated during experimental infection.

**Results:**

Although band-to-segmented neutrophils ratio became much higher (3.5) in infected pigs than in control group (0.3), marked neutropenia and lymphopenia were detected from 2 to 3 days post-infection. In addition to band neutrophils, the high number of other immature white blood cells, such as metamyelocytes, was observed during the course of infection. From the beginning of infection, atypical lymphocytes, with altered nuclear shape, arose and became 15% of total cells in the final phase of infection. Image scanning cytometry revealed hyperdiploid DNA content in atypical lymphocytes only from 5 days post-infection, indicating that DNA synthesis in pathological lymphocytes occurred in the later stages of infection.

**Conclusion:**

From this study, it can be concluded that ASFV infection leads to serious changes in composition of white blood cells. Particularly, acute ASFV infection in vivo is accompanied with the emergence of immature cells and atypical lymphocytes in the host blood. The mechanisms underlying atypical cell formation remain to be elucidated.

## Background

African swine fever virus (ASFV) is a large, enveloped virus containing double-stranded DNA (approximately 190 kilobase pairs) and the solo member of the recently created *Asfarviridae *[1]. It is the causative agent of a devastating hemorrhagic fever of domestic and wild swine. Depending on viral and host factors, ASFV infection of domestic swine can be accompanied with several disease forms, ranging from highly lethal (up to 100%) to subclinical. Infected pigs suffer from fever and anorexia as well as cyanosis of the skin, increased heart and respiratory rate and, finally, death. Currently, there is no vaccine or disease control strategy against ASFV other than movement restrictions, biosecurity and animal slaughter. Thus, the outbreaks of ASFV are still a great challenge for swine breeding.

ASFV shares some similarities in genome structure and replication strategies with other families of large, nucleocytoplasmically replicating DNA viruses, including *Iridoviridae*, *Mimiviridae*, *Poxviridae *and *Phycodnaviridae *[1,2]. These DNA viruses either replicate only in the cytoplasm, or begin their replication cycle in the nucleus and complete it in the cytoplasm of infected cells. Although ASFV has been considered exclusively a cytoplasmic virus, some authors have shown evidence of an early stage of viral replication in the nucleus [3,4]. More recently, it has been shown that the effect of ASFV infection on the nucleus is much more severe than previously believed, since the disassembling of lamina network and the redistribution of nuclear proteins, such as nucleophosmin and RNA polymerase II, has been observed from 4 hours post-infection [5]. Taken together, these findings clearly show a more serious involvement of the nucleus during ASFV infection, and therefore nuclear changes caused by ASFV can be of particular interest for future studies.

Although cellular receptors of viral entry are not clearly defined, it is well known that the primary cell types infected by ASFV belong to the mononuclear-phagocytic system, including macrophages and specific lineages of reticular cells [6,7]. The ability of ASFV to replicate in the mononuclear-phagocytic system seems critical in viral virulence. However, while several ASFV genes have been shown to be associated with host range and viral pathogenesis, much remains unclear about how ASFV induces cellular changes in the mononuclear-phagocytic system or whether the target cells belong only to the mononuclear-phagocytic system.

In our previous study we described atypical lymphocytes in primary culture of porcine bone marrow during ASFV infection in vitro [8]. The cytometry of atypical cells revealed increased DNA content, indicating that DNA synthesis occurred in atypical lymphocytes [8]. Consequently, we asked whether atypical lymphocytes arise during in vivo infection. Here we investigate the changes in population of porcine peripheral white blood cells over the course of ASFV infection in vivo. We show that acute viral infection of domestic swine results in the formation of atypical lymphocytes from the early stages of infection. Furthermore, image scanning cytometry of the cellular DNA content clearly shows that DNA synthesis occurs in atypical lymphocytes, indicating that it is common phenomenon for ASFV infection in vitro and in vivo.

## Methods

### Animal experiment and viral stock

In our study, fourteen healthy pigs of the same age (3-mo-old) and weight (40 kg) were used for infection and control. Nine pigs were infected by intramuscular injection and five pigs were used as the uninfected control. Animal care and euthanasia were done according to the AVMA Guidelines on Euthanasia, and local guideline for animal care and use (Institutional Review Board/Independent Ethics Committee of the Institute of Molecular Biology of NAS, IRB00004079). Carbon dioxide inhalation (75-80% carbon dioxide for 20 minute) was used to euthanatize infected animals after 7 days post-infection (dpi). Infections were carried out using ASFV (genotype II) distributed in the Republic of Armenia and the Republic of Georgia [9]. The titer of ASFV for each intramuscular injection was 10^4 ^50% hemadsorbing doses (HAD_50_)/ml. Virus titration was done as described previously and expressed as log_10 _HAD_50_/ml for non-adapted cells [10].

### Blood smears, Giemsa staining and white blood cells analysis

Peripheral blood was sampled from the ophthalmic venous sinus of either infected or uninfected pigs as described previously [11]. Fresh blood was used in preparing the blood smears by routine methods. For white blood cells analysis, slides were fixed in pure methanol and stained by Giemsa modified solution (azure B/azure II, eosin and methylene blue) according to the manufacturer's protocol (Sigma-Aldrich). White blood cells were examined under the light microscope at 1250 × magnification in a random sequence. At least 200 white blood cells in each sample were classified. The evaluation of cells and their sizes based on morphologic characteristics was described previously [8].

### Image scanning cytometry

For image scanning cytometry and DNA measurement, blood slides were fixed in 96% ethanol for 30 minutes and stained in fresh Schiff's reagent (DNA hydrolysis in 5 N hydrochloric acid for 60 minute at 22°C) by the method of Feulgen [12]. In order to measure DNA content (in conventional units) by image scanning cytometry, computer-equipped microscope-cytometer SMP 05 (OPTON) was used at 575 nm wavelength and at 1250 × magnification. Before the scanning process, each nucleus was contoured, and cytometry of nuclear DNA content of all studied types of cells were carried out at 1 to 7 dpi.

### Ploidy of cells

DNA content was expressed on a "c" scale, in which 1 c is the haploid amount of nuclear DNA occurred in normal (non-pathologic) diploid populations in G_0_/G_1_. The DNA content of unstimulated swine lymphocytes was used as a diploid standard for measurements. DNA measurements identify nuclei as aneuploid if they deviate more than 10% from 2 c, 4 c, 8 c, or 16 c; i.e. if they are outside of 2 c ± 0.2, 4 c ± 0.4, 8 c ± 0.8, or 16 c ± 1.6 values. The total number of cells in euploid areas of the DNA histogram rescaled by the mean corrective factor (1.8 c-2.2 c, 3.6 c-4.4 c, 7.2 c-8.8 c, and 14.4 c-17.6 c) was also calculated. The variability of DNA content in unstimulated lymphocytes did not exceed 10%.

### Statistics

White blood cell counts were performed in triplicate. At least 600 white blood cells (in triplicate for each pig) were observed for classification and DNA analysis. Data on blood cell counts were evaluated by a statistical test for pairwise comparisons in one-way ANOVA with unequal group variances (Tamhane's T2). Differences between control and infection were considered significant at *P *< 0.05. Results are expressed as mean of the percentage of each cell type ± standard deviation.

## Results

### Experimental infection

**Figure 1:**
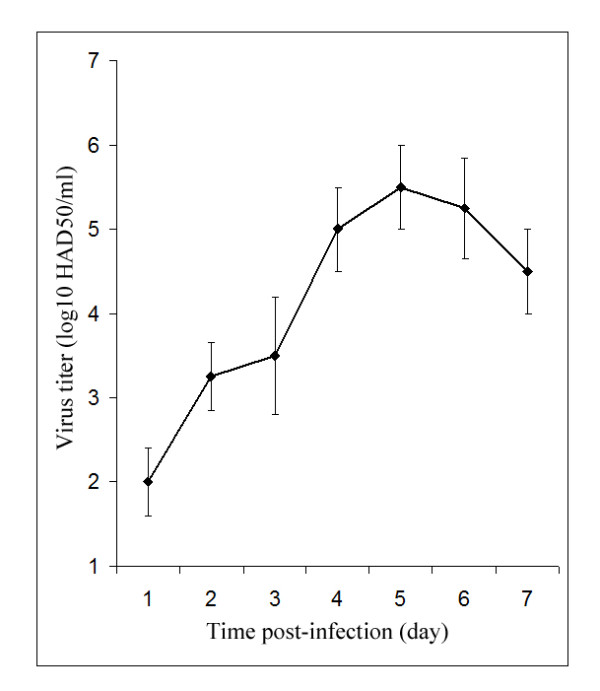
**ASFV titer during acute infection in swine**. Virus yields at different times post-infection (X axis) are expressed as log_10 _HAD_50_/ml (Y axis). Each point represents the mean of viral titer. Error bars indicate the standard errors.

Although ASFV isolates from different regions are capable of causing infections from highly acute to subacute, in our study, the clinical signs of experimental infection were not different from those in cited research with Malawi' 83 ASFV isolate [13]. The infected animals did not show changes in behavior caused by experimental infection and conditions. The first clinical signs were observed at 3 dpi when all infected pigs demonstrated loss of appetite and slight diarrhea. From 3 to 4 dpi, infected animals displayed hyperthermia with body temperature more than of 41°C. Simultaneously, decreased activity in behavior, difficulties in breathing and reddening of the skin were detected. Blood diarrhea and lethargy were seen at 5-6 dpi, and therefore all infected animals were sacrificed according to guidelines at 7 dpi. Although infected animals were asymptomatic up to 3 dpi, viremia appeared from 1-2 dpi and peaked on 5 dpi (viremia titers were 5.0-5.25 log_10 _HAD_50_/ml). The high titers of ASFV were determined in all pigs up to 7 dpi (Figure 1).

### Changes in peripheral white blood cells

Changes observed in the peripheral white blood cells of pigs infected with ASFV are summarized in table 1. The percent of fifteen cell types based on observations of 600 total cell counts was calculated during ASFV infection. Marked changes in the percent of cells were observed from 1 dpi (Table 1).

**Table 1:** Composition of white blood cells during acute ASFV infection in swine.

Cell types	The percent (%) of cells								ANOVA
		
	Control	1 dpi	2 dpi	3 dpi	4 dpi	5 dpi	6 dpi	7 dpi	p
Lymphoblasts	0	0	0.5 ± 0.1	3.2 ± 0.9**	3.6 ± 0.9**	18.9 ± 4.8**	10.3 ± 2.7**	2.8 ± 0.7**	< 0.001

Small lymphocytes	34.2 ± 7.6	18.6 ± 4.3*	25.7 ± 7.0	12.6 ± 3.5*	11.2 ± 2.9*	8.5 ± 2.6*	8.0 ± 2.0*	3.3 ± 1.0*	< 0.001

Medium lymphocytes	13.2 ± 3.5	10.4 ± 2.2*	10.6 ± 2.3*	3.8 ± 0.8*	7.1 ± 1.8	7.2 ± 2.1	8.4 ± 2.2	3.7 ± 0.8*	< 0.001

Large lymphocytes	8.5 ± 3.1	11.0 ± 2.1	6.4 ± 1.2	6.3 ± 1.9	5.3 ± 1.1	5.4 ± 1.2*	6.5 ± 1.9	6.5 ± 1.2	< 0.001

Reactive lymphocytes	0	6.1 ± 2.0**	3.7 ± 0.8**	3.8 ± 0.9**	8.0 ± 2.0**	4.5 ± 1.1**	4.7 ± 1.1**	14.8 ± 2.9**	< 0.001

Atypical lymphocytes	0	4.3 ± 1.1**	8.0 ± 1.8**	7.0 ± 1.9**	4.4 ± 1.0**	5.4 ± 1.2**	10.3 ± 2.8**	14.8 ± 3.3**	< 0.001

Monoblasts	0.5 ± 0.1	3.0 ± 0.4**	3.7 ± 0.6**	3.8 ± 1.1**	7.1 ± 2.1**	2.7 ± 0.8	2.8 ± 0.5*	0.2 ± 0.1	< 0.001

Monocytes	7.7 ± 2.8	7.3 ± 2.1	8.5 ± 1.3	10.8 ± 2.2	9.7 ± 2.9	9.0 ± 2.3	7.5 ± 1.6	7.4 ± 1.4	< 0.001

Metamyelocytes	0	1.2 ± 0.3**	3.7 ± 0.9**	3.8 ± 1.0**	6.2 ± 1.4**	6.3 ± 1.5**	7.5 ± 1.9**	7.3 ± 2.0**	< 0.001

Band neutrophils	7.7 ± 3.0	12.8 ± 3.4**	14.9 ± 2.5**	20.3 ± 3.4**	12.4 ± 3.0	4.5 ± 1.1*	5.6 ± 1.2	5.6 ± 1.8	< 0.001

Segmented neutrophils	23.1 ± 5.5	19.5 ± 4.0*	5.3 ± 0.9*	8.9 ± 1.5*	3.5 ± 0.9*	3.6 ± 0.9*	3.7 ± 0.8*	1.9 ± 0.3*	< 0.001

Eosinophils	4.9 ± 1.1	4.9 ± 1.3	1.6 ± 0.2*	3.8 ± 0.8	1.8 ± 0.5*	3.6 ± 0.7	2.8 ± 0.6	1.9 ± 0.2*	< 0.001

Basophils	0.2 ± 0.01	0.3 ± 0.02	0.4 ± 0.02	0.1 ± 0.02	0.3 ± 0.1	0.5 ± 0.1	0.4 ± 0.1	0.4 ± 0.1	< 0.001

Plasmocytes	0	0	1.6 ± 0.3**	3.2 ± 0.5**	2.7 ± 0.7**	3.6 ± 0.9**	4.7 ± 0.9**	4.6 ± 1.1**	< 0.001

Dead cells	0	0.6 ± 0.1	5.3 ± 1.0**	8.9 ± 1.6**	16.8 ± 3.4**	16.2 ± 4.0**	16.8 ± 3.8**	24.8 ± 4.8**	< 0.001

Control represents the mean of given cell type for each time point of infection

* Significant decrease compared with control (*p *< 0.05-*p *< 0.001)

** Significant increase compared with control (*p *< 0.05-*p *< 0.001)

**Figure 2:**
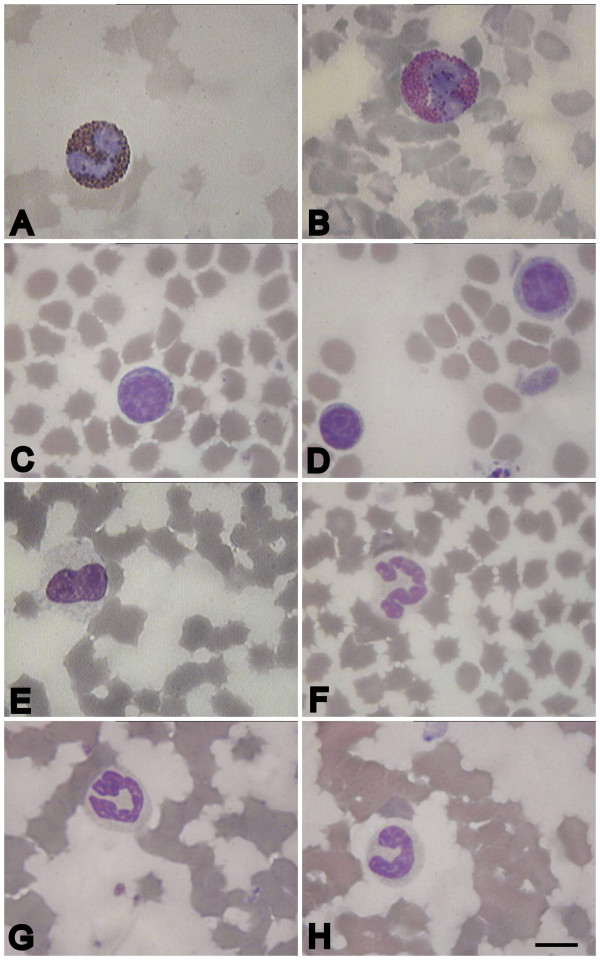
**Peripheral white blood cells of uninfected pigs**. A - eosinophil, B - basophil, C - medium lymphocyte, D - small and large lymphocytes, E - monocyte, F - segmented neutrophil, G, H - band neutrophils. Blood slides were stained by Giemsa modified solution. Cells were examined under the light microscope at 1250 × magnification. Scale bar is 10 μm.

The peripheral white blood of uninfected pigs mainly consisted of lymphocytes, band and segmented neutrophils as well as monocytes (Figure 2). The number of basophils remained low in both uninfected and infected pigs. Whereas band-to-segmented neutrophils ratio (B:S ratio) was about 0.65 at 1 dpi (compared with 0.3 in control), B:S ratio became much higher (3.5) at 4 dpi, showing an increased percentage of band neutrophils in population. However, marked lymphopenia and neutropenia (mature forms), with 2.5-fold reduction of lymphocytes and neutrophils at 7 dpi, were detected from 2 to 3 dpi.

**Figure 3:**
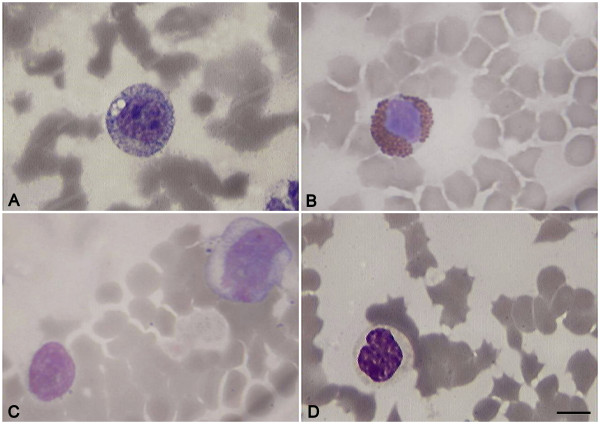
**Immature white blood cells during acute ASFV infection**. A - lymphoblast, B - eosinophilic metamyelocyte, C - monoblast, D - metamyelocyteBlood slides were stained by Giemsa modified solution. Cells were examined under the light microscope at 1250 × magnification. Scale bar is 10 μm.

**Figure 4:**
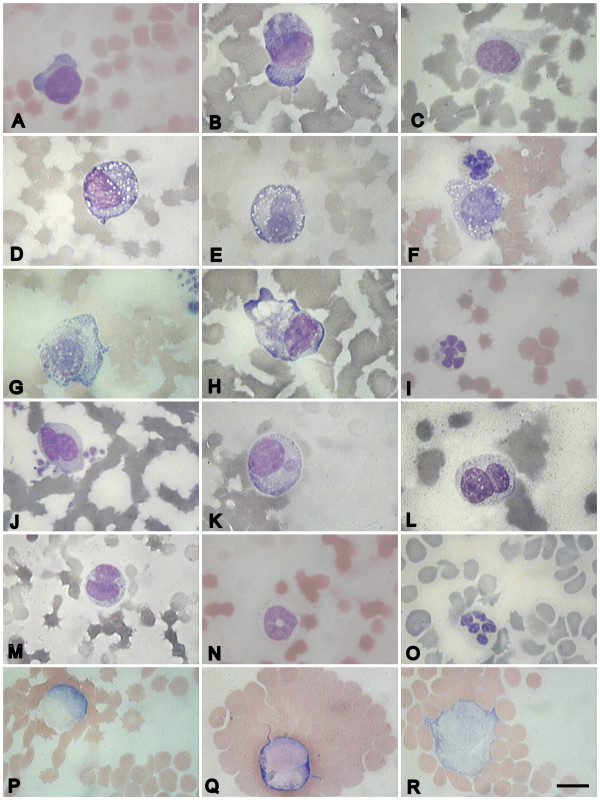
**Pathological and non-pathological white blood cells during acute ASFV infection**. A, B, C - different forms of reactive lymphocytes, D, F, G - reactive lymphoblasts with vacuoles, E, H - reactive monoblasts with vacuoles, I - plasmocyte, J, K, L, M - atypical lymphocytes with altered nuclear shape, N - atypical lymphocyte, O - hypersegmented neutrophil, P, Q, R - hemagglutination or hemadsorbtion. Blood slides were stained by Giemsa modified solution. Cells were examined under the light microscope at 1250 × magnification. Scale bar is 10 μm.

From the early stages of infection, immature forms of white blood cells, such as lymphoblasts, monoblasts and metamyelocytes (Figure 3), as well as atypical and reactive lymphocytes appeared (Figure 4) in the blood of infected pigs (Table 1). The number of lymphoblasts reached its peak (18.9% of the total cells) at 5 dpi and sharply decreased in the final phase of infection. The number of atypical and reactive lymphocytes increased throughout the entire period of infection and reached its maximal value in the premortal stage. ASFV infection caused a decrease in the number of small and medium-sized lymphoctyes, whereas the number of large-sized lymphocytes decreased but was not statistically significant (Table 1). By the last day of infection the percent of dead cells reached 24.5%, and the remaining cells were represented mainly by atypical and reactive lymphocytes as well as monocytes and metamyelocytes.

### Ploidy of lymphoblasts, monoblasts and atypical lymphocytes

**Figure 5:**
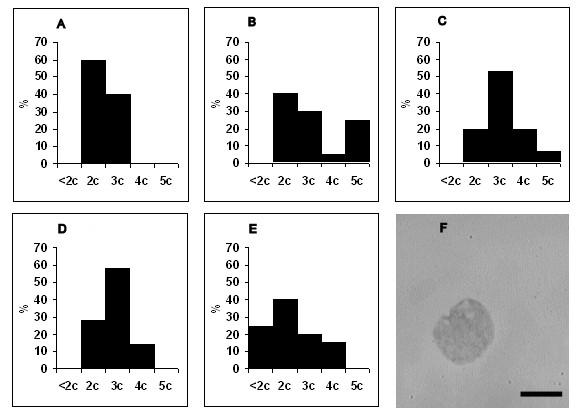
**Distribution of lymphoblasts by ploidy during acute ASFV infection**. A - ploidy of lymphoblasts at 3 dpi, B - at 4 dpi, C - at 5 dpi, D - at 6 dpi, E - at 7 dpi, F - the nucleus of lymphoblast stained by the method of Feulgen for ploidy analysis. X axis is the ploidy of cells. Y axis is the percent of distribution of cells. Data are presented from 3 dpi because no changes in ploidy (they were diploid as in control) were seen at 1-3 dpi. Cells were examined at 1250 × magnification. Scale bar is 10 μm.

**Figure 6:**
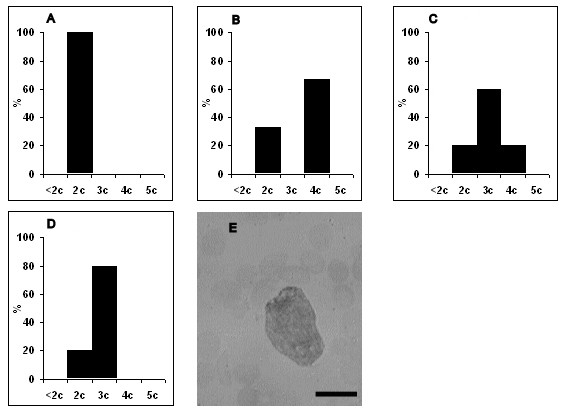
**Distribution of monoblasts by ploidy during acute ASFV infection**. A - ploidy of monoblasts at 3 dpi, B - at 4 dpi, C - at 5 dpi, D - at 6 dpi, E - the nucleus of monoblast stained by the method of Feulgen for ploidy analysis. X axis is the ploidy of cells. Y axis is the percent of distribution of cells. Data are presented from 3 dpi because no changes in ploidy (they were diploid as in control) were seen at 1-3 dpi. A few monoblasts, with hypodiploid DNA content, were detected at 7 dpi, and therefore data for 7 dpi are not presented. Cells were examined at 1250 × magnification. Scale bar is 10 μm.

**Figure 7:**
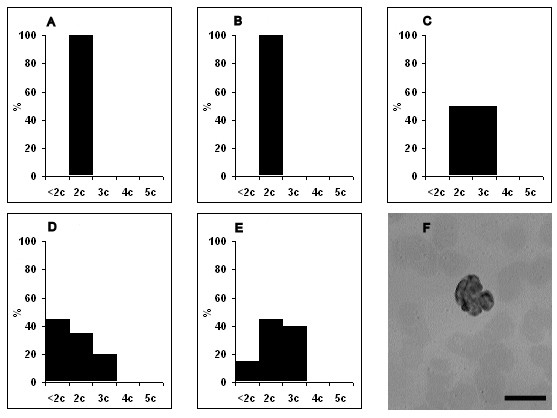
**Distribution of atypical lymphocytes by ploidy during acute ASFV infection**. A - ploidy of atypical lymphocytes at 3 dpi, B - at 4 dpi, C - at 5 dpi, D - at 6 dpi, E - at 7 dpi, F - the nucleus of lymphoblast stained by the method of Feulgen for ploidy analysisX axis is the ploidy of cells. Y axis is the percent of distribution of cells. Data are presented from 3 dpi because no changes in ploidy (they were diploid as in control) were seen at 1-3 dpi. Cells were examined at 1250 × magnification. Scale bar is 10 μm.

No changes in ploidy of lymphoblasts, monoblasts and atypical lymphocytes were seen at 1-3 dpi, and those cells were generally diploid (data not shown). Our data showed that the percent of diploid cells markedly reduced from 3 dpi. Also our data demonstrated that detected lymphoblasts did not belong to reactive lymphocytes subset due to the ploidy status of lymphoblasts [14]. The first lymphoblasts with hyperdiploid DNA content arose at 3 dpi, and became 60% of all lymphoblasts at 6 dpi (Figure 5). Unlike lymphoblasts, hyperdiploid monoblasts appeared at 5 dpi, and made up 80% of all monoblasts at 6 dpi (Figure 6). By 5 dpi half of the atypical lymphocytes were represented by hyperdiploid cells whose percent slightly decreased at 7 dpi (Figure 7). Whereas hypodiploid cells (< 2 c) were seen in the population of lymphoblasts and atypical lymphocytes in the later stages of infection, tetraploid cells were detected from 4 dpi. No atypical lymphocytes with tetraploid DNA content were detected during ASFV infection.

## Discussion

Since it has been shown that highly virulent ASFV strains target the mononuclear-phagocytic system, including macrophages, and destruct lymphoid tissues, it would be hypothesized that several other forms of white blood cells, such as lymphocytes, might be sensitive to ASFV infection. Indeed, it has been noted that highly virulent isolates of ASFV induce massive lymphopenia in vivo and in vitro [13,15-18]. The factors that are responsible for lymphopenia and the precise mechanism of lymphocyte apoptosis are still unknown. However, it has been suggested that tumor necrosis factor-alpha and interleukin-1 act as cytotoxic factors and their high expression is implicated in lymphopenia [19]. In addition to lymphopenia, we have shown that acute ASFV infection in vitro leads to the emergence of atypical lymphocytes, with abnormal nuclear shape and DNA content, indicating that ASFV may induce the formation of atypical cells in vivo [8].

This paper describes the changes in population of peripheral white blood cells of infected pigs. We observed that the infection of healthy pigs with ASFV led to profound changes in blood composition [20]. Particularly, the number of white blood cells significantly decreased from 3 dpi, coinciding with lymphopenia caused by apoptosis of lymphocytes and necrosis of lymphoid tissues [15-18]. Herein we observed a band-to-segmented neutrophils ratio that became 3.0 (left shift) at the later stages of infection, indicating the emergence of immature neutrophils in blood. Some anomalies, such as impaired nuclear segmentation of mature neutrophilic granulocytes, mimic shift of neutrophils to the left [21]. However, metamyelocytes were detected only in the blood of infected pigs, suggesting that the high number of immature neutrophils was a particular consequence of left shift and not a result of mimicry.

Other immature cells, such as lymphoblasts and monoblasts, were also observed in the blood of ASFV-infected pigs. Our data indicate that ASFV infection directly or indirectly influences on releasing of lymphoblasts and monoblasts from the bone marrow. The emergence of immature cells in peripheral blood can be attributed to activation of hematopoiesis in bone marrow. Although increased hematopoiesis might exert a positive influence on the host response to acute infection, the hematologic data obtained in this and other studies suggest that ASFV infection leads to impaired hematopoiesis [13]. Cytometry of lymphoblasts and monoblasts revealed redundant DNA content from 3-4 dpi. This observation accords with previous studies where it was noted that some immature white blood cells had hyperdiploid DNA content [22,23]. The final phase of the disease was characterized not only by hyperdiploid cells but also by lymphoblasts in which DNA content was less than in diploid cells (i.e. hypodiploid). This phenomenon might be explained by degradation of DNA due to apoptosis of lymphocytes [16,19,24,25].

In addition to the previous study, when we detected atypical lymphocytes in primary culture of bone marrow during ASFV infection, the current study shows that atypical lymphocytes also arise as a consequence of in vivo infection. Shiftan and Mendelsohn pointed out the criteria that can be used for definition of atypical lymphocytes [26]. One of the criteria is the ability of atypical lymphocytes to synthesize DNA, whereas non-pathologic lymphocytes are disabled to do so. Although atypical lymphocytes were observed during the entire course of infection, hyperdiploid DNA content occurred in the final phase of infection. Thus, we can hypothesize that atypical lymphocytes detected at early days of infection were transient forms that became atypical after DNA synthesis [27]. The precise mechanism for DNA synthesis remains to be elucidated, but our working hypothesis is that ASFV directly or indirectly influences the mitotic activity of lymphocytes, leading to incomplete mitosis. This hypothesis is supported by the experiments demonstrating that other DNA viruses, such as human papillomavirus type 1, are able to induce S phase entry in cells that would otherwise be in G0 as well as arrest at G2/M [28]. Therefore, it would be interesting to carry out a series of experiments to determine whether ASFV interferes in the cell cycle of lymphocytes or lymphoblasts.

## Conclusion

In conclusion, we have shown that infection of pigs with ASFV results in changes in the composition of white blood cells. Although lymphopenia and neutropenia are observed, the number of some white blood cells, such as band neutrophils and metamyelocytes, increases during the course of infection. The emergence of immature cells in peripheral blood can be explained by the activation of hematopoiesis that fails in replacement of dead cells by mature forms. Beside immature cells, atypical lymphocytes are also detected in infected pigs. Since we previously reported that atypical lymphocytes arise during in vitro infection, and since the same phenomenon is demonstrated here for in vivo infection, we conclude that acute ASFV infection is accompanied with the formation of atypical lymphocytes in domestic pigs.

## Competing interests

On behalf of all co-authors, the corresponding author declares that they have no competing interests.

## Authors' contributions

ZK planned the study and drafted the manuscript, HZ participated in blood analysis and drafted the manuscript with ZK, HA prepared slides and carried out blood analysis, KS and HV helped the animal experiment, LH carried out cytochemical stains, LA and AA carried out the cytometry of cells, EK performed statistical analysis and participated in study design. All authors read and approved the final version of manuscript.
